# Uncovering the Cardiovascular Threat: A Comprehensive Examination of Liver Fibrosis and Subclinical Atherosclerosis in Non-alcoholic Fatty Liver Disease

**DOI:** 10.7759/cureus.46946

**Published:** 2023-10-13

**Authors:** Niketa Sharma, Swarupa Chakole, Bhushan Wandile

**Affiliations:** 1 Medicine, Jawaharlal Nehru Medical College, Datta Meghe Institute of Higher Education and Research, Wardha, IND; 2 Community Medicine, Jawaharlal Nehru Medical College, Datta Meghe Institute of Higher Education and Research, Wardha, IND

**Keywords:** lifestyle interventions, multidisciplinary care, non-invasive diagnostics, atherosclerosis, liver fibrosis, nafld

## Abstract

Non-alcoholic fatty liver disease (NAFLD) has emerged as a global epidemic intricately linked to the rising tide of obesity and metabolic syndrome. This comprehensive review delves into the complex web of relationships between NAFLD, liver fibrosis, and subclinical atherosclerosis, shedding light on their interplay, shared risk factors, and clinical implications. NAFLD encompasses a spectrum of liver conditions, from the benign non-alcoholic fatty liver (NAFL) to the more severe non-alcoholic steatohepatitis (NASH), characterized by inflammation and hepatocellular injury. Central to the discussion is the insidious development of liver fibrosis, the ominous harbinger of progressive liver damage, cirrhosis, and hepatocellular carcinoma. The increasing prevalence of NAFLD, now affecting a quarter of the global population, poses a significant public health challenge. Its association with obesity, insulin resistance, and metabolic syndrome highlights the multifactorial nature of this disease. However, NAFLD's repercussions extend beyond the liver. This review unveils a potent connection between NAFLD and subclinical atherosclerosis, the early precursor to cardiovascular disease. Individuals with NAFLD face an elevated risk of atherosclerosis, even without traditional cardiovascular risk factors. The intricate link between these two conditions is illuminated through shared pathophysiological pathways, including systemic inflammation, insulin resistance, and dyslipidemia. Understanding the interplay between liver fibrosis and subclinical atherosclerosis has profound clinical implications. Patients with advanced fibrosis or cirrhosis are not only at risk of liver-related complications but also of cardiovascular events. This necessitates a holistic approach to patient care, with lifestyle modifications and pharmacological interventions simultaneously managing both conditions. Physicians must prioritize early detection and collaborate across disciplines to provide comprehensive care. Looking ahead, the future holds promising avenues of research. Emerging areas include genetics and precision medicine, microbiome research, and epigenetics, which may unveil new therapeutic targets. Innovations in diagnostics and therapeutics, such as non-invasive biomarkers and combination therapies, offer hope for more effective management. Long-term outcomes and survivorship research will provide insights into the lasting impact of interventions. In conclusion, this review underscores the imperative of addressing liver fibrosis and atherosclerosis in the context of NAFLD. It is a call to action for healthcare professionals, researchers, and policymakers to work collaboratively, promote early detection, and advance our understanding of these interconnected conditions. By doing so, we can enhance patient outcomes and chart a course toward a healthier future for those grappling with NAFLD and its intricate web of consequences.

## Introduction and background

Non-alcoholic fatty liver disease (NAFLD) has recently become a significant public health concern. It represents a spectrum of liver disorders characterized by fat accumulation in hepatocytes without excessive alcohol consumption. NAFLD has become a global epidemic, affecting millions of individuals worldwide, and its prevalence continues to rise in parallel with the obesity and metabolic syndrome epidemics. While initially recognized as a hepatic condition, the implications of NAFLD extend far beyond the liver. This review explores the intricate relationship between NAFLD, liver fibrosis, and cardiovascular health, shedding light on the complex interplay between these seemingly distinct entities [1,2]. Once considered a benign condition, NAFLD has evolved into a significant public health issue due to its potential progression to more severe liver-related complications, such as cirrhosis and hepatocellular carcinoma. Characterized by the accumulation of triglycerides in hepatocytes, NAFLD encompasses a range of histological features, from simple steatosis (non-alcoholic fatty liver, NAFL) to non-alcoholic steatohepatitis (NASH), the latter of which is associated with inflammation and fibrosis [1]. The prevalence of NAFLD is closely tied to the rising tide of obesity, sedentary lifestyles, and unhealthy dietary habits in many parts of the world. It is estimated that approximately 25% of the global population has NAFLD, with higher rates in Western countries. In some high-risk populations, such as those with type 2 diabetes and morbid obesity, the prevalence exceeds 70%. The economic burden associated with NAFLD is substantial, stemming from direct healthcare costs and the indirect costs of lost productivity and disability [3]. Beyond its impact on liver health, NAFLD has emerged as a harbinger of systemic disease, particularly cardiovascular disease (CVD). Research has revealed a striking association between NAFLD and increased cardiovascular risk. Individuals with NAFLD are more likely to develop atherosclerosis, experience cardiovascular events, and exhibit features of metabolic syndrome, including insulin resistance, dyslipidemia, and hypertension. This connection between NAFLD and cardiovascular health extends to liver fibrosis within NAFLD patients [4]. This comprehensive review explores the intricate relationship between NAFLD, liver fibrosis, and cardiovascular health. We will examine the mechanistic underpinnings connecting these conditions and explore the clinical implications for patients. Furthermore, we will discuss current diagnostic methods and treatment options for NAFLD and liver fibrosis while considering their impact on cardiovascular outcomes. Finally, we will identify gaps in current knowledge and propose directions for future research in this crucial area.

## Review

Non-alcoholic fatty liver disease

Definition and Classification of NAFLD

NAFLD constitutes a heterogeneous group of liver disorders marked by excessive fat accumulation within hepatocytes. Unlike alcoholic liver disease, NAFLD manifests in individuals who consume little or no alcohol, making it a distinct entity. This condition represents a spectrum of liver abnormalities with varying degrees of severity. At its mildest end, we find NAFL, characterized by hepatic steatosis, or fat accumulation within liver cells, without inflammation or hepatocellular injury. Significantly, NAFL is generally associated with a more favorable prognosis [1]. On the other hand, NASH represents a more severe form of NAFLD. NASH is identified by hepatic steatosis accompanied by inflammation and hepatocellular injury. It is a condition that warrants significant attention due to its potential to progress to more dire stages of liver disease, including fibrosis, cirrhosis, and, in extreme cases, hepatocellular carcinoma. The dichotomy between NAFL and NASH underscores the importance of accurate diagnosis and risk stratification within the realm of NAFLD, as it significantly influences patient management and clinical outcomes [1].

Prevalence and Epidemiology

NAFLD has emerged as a widespread global health concern, impacting approximately a quarter of the world's population. The estimated global prevalence of NAFLD among adults is 32%, which is higher among males (40%) than females (26%). This alarming prevalence positions NAFLD among the most common liver diseases on a global scale. However, there are substantial regional disparities in its occurrence. It is notably more prevalent in Western nations, particularly North America and Europe. These regional variations are closely linked to lifestyle factors prevalent in these areas, including high-calorie diets, sedentary lifestyles, and the ongoing obesity epidemic [5]. Obesity plays a central role as a key risk factor for developing NAFLD. As the worldwide obesity crisis continues to escalate, the incidence of NAFLD is expected to rise in tandem. The strong connection between NAFLD and obesity highlights the urgency of addressing this intertwined public health issue [6]. While NAFLD affects individuals of all genders, it exhibits a slightly higher prevalence in males. Remarkably, NAFLD is not confined by age; it can manifest at any stage of life, including in children. Nevertheless, its prevalence significantly increases with advancing age, emphasizing the importance of early detection and intervention across all age groups [7].

Risk Factors and Comorbidities

Obesity: Excessive body weight, particularly visceral adipose tissue accumulation, is one of the most robust risk factors for NAFLD. This connection is underpinned by the dysfunction of adipose tissue and the onset of insulin resistance, which contribute to the development and progression of NAFLD [6].

Insulin resistance: Insulin resistance is a central pathophysiological feature of NAFLD. It characterizes the impaired ability of cells to respond to insulin and plays a pivotal role in the condition's genesis. Moreover, insulin resistance often coexists with other metabolic disturbances, including metabolic syndrome and type 2 diabetes, further complicating the clinical picture [8].

Dyslipidemia: Aberrations in lipid profiles are commonplace among NAFLD patients. Elevations in triglycerides and low high-density lipoprotein (HDL) cholesterol levels are frequently observed. This dyslipidemia contributes to the intricate interplay between NAFLD and cardiovascular risk factors [9].

Hypertension: High blood pressure is another prevalent comorbidity in individuals with NAFLD. Its presence heightens the cardiovascular risk in these patients and underscores the need for comprehensive cardiovascular evaluation and management [10].

Genetics: While lifestyle factors play a significant role in the development of NAFLD, genetic predisposition is increasingly recognized. Specific genetic polymorphisms related to lipid metabolism, inflammation, and insulin signaling pathways have been implicated in the susceptibility to NAFLD and its progression. Understanding the genetic underpinnings of NAFLD can aid in risk assessment and personalized management [11].

Diagnostic Methods and Assessment

Liver function tests: Serum markers such as alanine transaminase (ALT) and aspartate transaminase (AST) are commonly used in clinical practice. Elevated levels of these enzymes often indicate liver damage, although they lack specificity for NAFLD [12].

Imaging studies: Non-invasive imaging techniques play a crucial role in NAFLD assessment. These include ultrasound, computed tomography (CT), and MRI. These imaging modalities can detect the presence and quantify the degree of hepatic steatosis (fat accumulation) and, in some cases, provide insights into fibrosis severity [13].

Liver biopsy: Despite its invasiveness, liver biopsy remains the gold standard for diagnosing NAFLD, distinguishing between NAFL and NASH, and assessing the extent of liver fibrosis. It allows for the histological evaluation of liver tissue, grading inflammation, and staging fibrosis. However, liver biopsy has limitations, including sampling variability and potential complications [14].

Non-invasive scoring systems: Several scoring systems and blood-based biomarkers have been developed to circumvent the need for liver biopsy. Examples include the NAFLD Fibrosis Score (NFS) and Fibrosis-4 Index (FIB-4). These tools utilize clinical and laboratory parameters to predict the presence and severity of liver fibrosis, aiding in risk stratification [15].

Imaging-based fibrosis assessment: Techniques like transient elastography (FibroScan) and magnetic resonance elastography (MRE) offer non-invasive assessments of liver stiffness, providing valuable information about the extent of fibrosis. These modalities are beneficial for monitoring disease progression and treatment response [16].

Liver fibrosis in NAFLD

Mechanisms of Liver Fibrosis Development in NAFLD

Inflammation: Chronic inflammation within the liver is recognized as a central driver of fibrosis in NAFLD. This inflammatory milieu arises from many factors, including the toxic effects of lipid accumulation (lipotoxicity) within hepatocytes, oxidative stress-induced cellular damage, and the release of pro-inflammatory cytokines from activated immune cells. These inflammatory responses create a microenvironment conducive to fibrogenesis [17]. Hepatic stellate cell (HSC) activation: HSCs play a pivotal role in developing liver fibrosis. In response to various stimuli, including inflammation and oxidative stress, quiescent HSCs can profoundly transform into myofibroblast-like cells. These activated HSCs become profibrogenic and are responsible for the excessive production and deposition of extracellular matrix (ECM) components, mainly collagen, in the liver. This aberrant ECM accumulation disrupts the normal liver architecture and function [18]. Oxidative stress: It is characterized by an imbalance between the production of reactive oxygen species (ROS) and the antioxidant defense mechanisms, which play a crucial role in hepatocellular injury and apoptosis. Mitochondrial dysfunction, often associated with NAFLD, contributes to the generation of ROS, which, in turn, exacerbates oxidative stress and cellular damage. These processes further amplify the fibrotic response [19]. Lipid metabolism dysregulation: Dysregulation of lipid metabolism represents a fundamental hallmark of NAFLD. It leads to the accumulation of lipids, mainly triglycerides, within hepatocytes. This intrahepatic lipid accumulation creates a lipotoxic environment characterized by lipids' toxic effects, promoting inflammation and fibrogenesis. Lipotoxicity directly injures hepatocytes and triggers immune responses, contributing to hepatic inflammation [20]. Endoplasmic reticulum (ER) stress: ER is a crucial organelle in protein synthesis and lipid metabolism. ER stress occurs when the organelle's capacity to fold proteins and handle lipids is overwhelmed, often due to protein misfolding and lipotoxicity. ER stress triggers cellular responses, including the unfolded protein response (UPR), that can contribute to the progression of liver fibrosis [21].

Stages of Fibrosis and Their Clinical Significance

Stage 0 (F0), no fibrosis: In this initial stage, marked as F0, patients exhibit what is known as simple steatosis, or NAFL, where there is an accumulation of fat within hepatocytes but no evidence of fibrosis. At this point, the liver's structural integrity remains largely intact, and patients generally have a favorable prognosis with a low risk of liver-related complications [21]. Stage 1 (F1), periportal or perisinusoidal fibrosis: Progressing to F1, minimal fibrosis is present, often localized to the periportal or perisinusoidal areas of the liver. Patients at this stage are still at a relatively low risk of liver-related complications, and the disease progression is typically gradual, allowing time for interventions and lifestyle modifications [22]. Stage 2 (F2), zone 3 perisinusoidal fibrosis with bridging fibrosis: F2 marks a notable advancement in fibrotic changes. In this stage, bridging fibrosis becomes evident, connecting adjacent portal tracts, but the liver has not yet reached the cirrhotic stage (F4). Patients with F2 fibrosis face a moderate risk of disease progression, emphasizing the importance of close monitoring and targeted interventions [23]. Stage 3 (F3), bridging fibrosis with architectural distortion: Transitioning to F3, bridging fibrosis becomes more extensive, leading to architectural distortion within the liver tissue. The liver's altered architecture significantly increases the risk of portal hypertension and clinical decompensation. Vigilant monitoring and management strategies become increasingly vital in this stage to prevent further liver damage [24]. Stage 4 (F4), cirrhosis: The ultimate and most advanced stage of liver fibrosis in NAFLD is F4, representing cirrhosis. The liver undergoes extensive fibrotic remodeling at this stage, characterized by regenerative nodules and substantial architectural changes. Patients with cirrhosis are at substantial risk of liver failure, hepatocellular carcinoma (HCC), and complications related to portal hypertension, including ascites, variceal bleeding, and hepatic encephalopathy. Management focuses on mitigating these risks and providing comprehensive care to improve overall outcomes [25].

Diagnostic Tools for Assessing Liver Fibrosis

Liver biopsy: Liver biopsy, despite its invasiveness, has long served as the gold standard for staging fibrosis and grading inflammation in NAFLD. This procedure provides detailed histological information about the extent of fibrosis and the presence of inflammatory changes. While it offers high diagnostic accuracy, its invasive nature is associated with risks and limitations, including sampling variability and the potential for complications [14]. Non-invasive tests: Recognizing the limitations of liver biopsy, non-invasive methods have gained prominence as alternatives for assessing fibrosis in NAFLD. These include blood-based biomarkers, such as FibroTest and the FIB-4, which utilize clinical and laboratory parameters to predict the presence and severity of fibrosis. These tests are relatively convenient and carry no risk of procedural complications, making them attractive options for patients [26]. Imaging-based techniques: Non-invasive imaging-based techniques offer valuable insights into liver fibrosis. Transient elastography, commonly known as FibroScan, employs ultrasound to measure liver stiffness, providing information about the degree of fibrosis. MRE is another advanced imaging modality that highly accurately assesses liver stiffness. Additionally, cross-sectional imaging techniques like computed tomography (CT) and MRI can indirectly assess liver fibrosis by evaluating liver stiffness and nodularity [27].

Management and Treatment Options for Liver Fibrosis in NAFLD

Lifestyle modification: Lifestyle changes are fundamental to managing NAFLD-related fibrosis. Weight loss, achieved through dietary modifications and increased physical activity, is a cornerstone of treatment. Losing excess weight can lead to significant improvements, including the regression of liver fibrosis. Lifestyle changes include adopting a balanced diet, limiting added sugars and saturated fats, and exercising regularly [28]. Pharmacological interventions: While no medications have received FDA approval for NAFLD-related fibrosis, several drugs have shown promise in clinical trials. These include vitamin E, which has demonstrated potential for reducing liver inflammation and fibrosis in certain patients. Pioglitazone, an insulin-sensitizing medication, has also been investigated for its ability to improve liver histology in NAFLD. Additionally, obeticholic acid, a farnesoid X receptor agonist, has shown benefits in select cases [29]. Treatment of comorbidities: Addressing comorbid conditions is integral to preventing the progression of liver fibrosis in NAFLD. Management of comorbidities such as diabetes, dyslipidemia, and hypertension is essential. Optimizing glycemic control, lipid profiles, and blood pressure can improve liver health [30]. Antifibrotic therapies: Several investigational antifibrotic agents are developing and are promising to treat advanced fibrosis in NAFLD. These emerging therapies target specific fibrogenic pathways and have the potential to slow or reverse fibrosis progression [31]. Monitoring and surveillance: Patients with advanced fibrosis or cirrhosis require ongoing monitoring and surveillance to detect complications promptly. Regular hepatocellular carcinoma (HCC) assessments, including imaging studies and serum biomarkers, are essential. Screening for esophageal varices and hepatic encephalopathy may also be necessary in cirrhotic patients [32].

Subclinical atherosclerosis in NAFLD

Overview of Atherosclerosis and Its Significance

Atherosclerosis, a multifaceted and progressive vascular condition, plays a pivotal role in the landscape of CVD. It is defined by the gradual accumulation of plaque within arteries, composed of lipids, inflammatory cells, and fibrous tissue. Over time, this plaque buildup leads to the narrowing and stiffening of affected blood vessels, disrupting normal blood flow and potentially causing clinical complications [33]. The significance of atherosclerosis cannot be overstated, primarily due to its intimate connection with CVD, a category of diseases that collectively represent the foremost cause of morbidity and mortality on a global scale. Key cardiovascular conditions linked to atherosclerosis include coronary artery disease, which can result in heart attacks; cerebrovascular disease, often leading to strokes; and peripheral artery disease, affecting blood flow to the extremities [34]. Atherosclerosis's pathogenic processes impair arteries' structural integrity and function and foster a proinflammatory environment, facilitating further plaque deposition and disease progression. This chronic and systemic nature underscores the need for comprehensive approaches to prevent, diagnose, and manage atherosclerosis and its associated cardiovascular consequences. The ongoing research into the mechanisms of atherosclerosis and the development of innovative diagnostic and therapeutic strategies promise to reduce the immense global burden imposed by CVD [35].

The Link Between NAFLD and Subclinical Atherosclerosis

Increased cardiovascular risk: NAFLD patients face a notably heightened risk of developing atherosclerosis and subsequent cardiovascular events, even when traditional CVD risk factors are not present or are well-controlled. This association highlights the independent contribution of NAFLD to cardiovascular risk [4]. Shared risk factors: NAFLD and atherosclerosis share a constellation of common risk factors. These include obesity, insulin resistance, dyslipidemia, and systemic inflammation, all of which play pivotal roles in the pathogenesis of both conditions. The interplay of these risk factors contributes to the development and progression of both NAFLD and atherosclerosis [36]. Proinflammatory state: NAFLD is characterized by chronic inflammation within the liver, and this proinflammatory state can extend systemically. The presence of inflammation in NAFLD is particularly relevant in atherosclerosis, as it may contribute to endothelial dysfunction, a key early event in developing atherosclerotic plaques. Endothelial dysfunction impairs the normal function of blood vessel walls, making them more prone to plaque formation and narrowing [37].

Mechanisms Underlying the Development of Atherosclerosis in NAFLD

Systemic inflammation: NAFLD is characterized by a proinflammatory state, with elevated levels of proinflammatory cytokines and adipokines produced by adipose tissue and the liver. This chronic systemic inflammation extends beyond the liver and can promote atherogenic processes. Inflammation contributes to endothelial dysfunction, oxidative stress, and the creation of a prothrombotic environment, all of which are conducive to the development of atherosclerosis [38]. Dyslipidemia: NAFLD is frequently associated with an unfavorable lipid profile, characterized by increased triglyceride levels and decreased HDL cholesterol levels. Additionally, individuals with NAFLD may have small, dense, low-density lipoprotein (LDL) particles more prone to atherogenesis. These lipid abnormalities contribute to the progression of atherosclerosis and increase the risk of cardiovascular events [39]. Insulin resistance: Insulin resistance, a hallmark of NAFLD, plays a pivotal role in the development of atherosclerosis. Insulin resistance impairs glucose metabolism and disrupts lipid metabolism, leading to dyslipidemia. Moreover, insulin resistance can directly affect endothelial function, exacerbating atherogenic processes [40]. NASH: In severe cases of NAFLD, particularly NASH, the liver may release hepatokines and damage-associated molecular patterns (DAMPs) into the circulation. These molecules can further propagate systemic inflammation and oxidative stress, contributing to the pathogenesis of atherosclerosis beyond the liver [41].

Diagnostic Methods for Assessing Subclinical Atherosclerosis

Carotid intima-media thickness (CIMT): CIMT measurement, typically performed via ultrasound, evaluates the thickness of the inner layers of the carotid artery wall. Increased CIMT indicates atherosclerosis and serves as a surrogate marker for cardiovascular risk. It is a non-invasive and cost-effective method [42]. Coronary artery calcium (CAC) Score: CT scans can quantify the amount of calcium deposits within the coronary arteries, generating a CAC score. This score correlates with the burden of atherosclerotic plaques within the coronary arteries. A higher CAC score is associated with an increased risk of cardiovascular events and provides valuable information for risk stratification [43]. Endothelial function testing: Techniques like flow-mediated dilation (FMD) assess the endothelium's function and the blood vessels inner lining. Impaired endothelial function is an early sign of atherosclerosis and is often observed before the development of overt clinical symptoms. FMD measures the ability of blood vessels to dilate in response to increased blood flow and is a non-invasive way to evaluate endothelial function [44]. Biomarkers: Blood-based biomarkers are used to assess the presence of inflammation and endothelial dysfunction associated with atherosclerosis. These biomarkers include high-sensitivity C-reactive protein (hs-CRP), which reflects systemic inflammation, as well as lipoprotein(a) and asymmetric dimethylarginine (ADMA), which are indicators of endothelial dysfunction. Elevated levels of these biomarkers are associated with an increased risk of atherosclerosis-related events [45]. Non-invasive imaging: Advanced imaging modalities, such as positron emission tomography (PET) and MRI, can visualize atherosclerotic plaques and assess their characteristics. These techniques provide valuable information about plaque composition, stability, and vulnerability to rupture, which are critical for risk assessment and treatment planning [46].

The interplay between liver fibrosis and subclinical atherosclerosis

Research Findings on the Relationship between Liver Fibrosis and Atherosclerosis

Cross-sectional studies: Numerous cross-sectional studies have provided compelling evidence of a positive association between the presence and severity of liver fibrosis in NAFLD patients and markers of subclinical atherosclerosis. These studies often utilize non-invasive assessments, such as CIMT measurements and CAC scores, to evaluate atherosclerotic burden. The findings consistently demonstrate that individuals with more advanced liver fibrosis tend to exhibit higher levels of subclinical atherosclerosis, emphasizing the close relationship between these conditions [47]. Longitudinal studies: Longitudinal studies have further reinforced the connection between liver fibrosis and atherosclerosis by revealing that the progression of liver fibrosis in NAFLD is intricately linked to the development and progression of atherosclerosis over time. These studies underscore the importance of ongoing monitoring for both conditions, as their trajectories can intersect and influence one another [48]. Shared risk factors: The relationship between liver fibrosis and atherosclerosis is buttressed by shared risk factors contributing to the development and progression of both conditions. These risk factors include obesity, insulin resistance, chronic inflammation, and dyslipidemia. These common pathways, such as inflammation and lipid dysregulation, create a favorable environment for the simultaneous emergence and progression of liver fibrosis and atherosclerosis in individuals with NAFLD [49].

Shared Risk Factors and Pathophysiological Pathways

Obesity: Obesity is a pivotal risk factor that plays a central role in liver fibrosis and subclinical atherosclerosis. Excess body weight, particularly visceral adiposity, promotes adipose tissue dysfunction, insulin resistance, and the secretion of proinflammatory adipokines. These processes collectively contribute to the development and progression of liver fibrosis within the context of NAFLD. Simultaneously, obesity-induced inflammation and metabolic derangements fuel atherogenic processes, laying the foundation for the initiation and progression of atherosclerosis [50]. Insulin resistance: It is a hallmark of obesity, and metabolic syndrome is a common thread linking liver fibrosis and atherosclerosis. In the liver, insulin resistance promotes the accumulation of lipids within hepatocytes, initiating the cascade of events that lead to hepatic steatosis and, eventually, liver fibrosis. Systemically, insulin resistance impairs endothelial function, marking the inception of atherosclerotic processes by disrupting glucose and lipid metabolism [51]. Dyslipidemia: Dyslipidemia in NAFLD encompasses an atherogenic lipid profile characterized by elevated levels of triglycerides, reduced HDL cholesterol, and the presence of small, dense LDL particles. These lipid abnormalities are a shared feature in individuals with NAFLD and contribute to the development of atherosclerosis. Small, dense LDL particles, in particular, are more prone to atherogenesis, facilitating the formation of atherosclerotic plaques [52]. Inflammation: Chronic inflammation is a common thread that ties liver fibrosis and atherosclerosis together. In NAFLD, hepatocellular injury and inflammation within the liver contribute to the systemic release of proinflammatory cytokines, creating a proinflammatory milieu that promotes endothelial dysfunction and oxidative stress. These systemic inflammatory processes contribute to the initiation and progression of atherosclerosis [53]. Oxidative stress: Oxidative stress is a shared pathophysiological mechanism in liver fibrosis and atherosclerosis. Mitochondrial dysfunction and excessive production of ROS lead to cellular damage, inflammation, and oxidative stress within the liver and the vasculature. These oxidative processes further fuel the development of both conditions [54].

Clinical Implications and Prognostic Significance

Cardiovascular risk assessment: Patients diagnosed with NAFLD and concurrent liver fibrosis should undergo a thorough cardiovascular risk assessment. This assessment is crucial because these individuals are at an elevated risk of experiencing atherosclerosis-related events, such as heart attacks and strokes, even without traditional cardiovascular risk factors. Recognizing this increased risk allows healthcare providers to implement appropriate preventive measures and interventions [55]. Prognostic significance: The coexistence of liver fibrosis and subclinical atherosclerosis can synergistically affect patient outcomes. Both conditions independently contribute to adverse health outcomes, and their simultaneous presence further amplifies the risk of cardiovascular events, liver-related complications, and overall mortality in individuals with NAFLD. Therefore, understanding and addressing this intricate relationship can profoundly impact patient prognosis [56]. Early intervention: Recognizing the interplay between liver fibrosis and atherosclerosis underscores the importance of early intervention and management. Detecting and addressing both conditions in their subclinical stages can help mitigate their progression and improve long-term outcomes. This may involve lifestyle modifications, pharmacological interventions, and managing shared risk factors, such as obesity, insulin resistance, and dyslipidemia. Early intervention strategies aim to reduce cardiovascular risk and prevent the advancement of liver fibrosis and its associated complications, such as cirrhosis and hepatocellular carcinoma [57].

Potential Therapeutic Strategies Targeting Both Conditions

Lifestyle modification: Lifestyle interventions are central in managing liver fibrosis and subclinical atherosclerosis. Weight loss through dietary changes and increased physical activity can improve insulin sensitivity, reduce liver fat, and mitigate the risk of atherosclerosis. Lifestyle modifications also have the potential to lower blood pressure, improve lipid profiles, and enhance overall cardiovascular health [58]. Pharmacological interventions: Medications that target common risk factors shared by both liver fibrosis and atherosclerosis can be beneficial. For instance, statins are commonly prescribed to address dyslipidemia and reduce the risk of cardiovascular events. Anti-diabetic agents, such as metformin or GLP-1 receptor agonists, may help improve insulin resistance and glycemic control, benefiting both liver and cardiovascular health. Careful consideration of medication regimens should consider the patient's individual risk factors and medical history [59]. Antifibrotic therapies: Developing antifibrotic therapies specific to NAFLD holds promise for halting or reversing liver fibrosis and reducing inflammation. As inflammation is a central driver of atherosclerosis, antifibrotic agents may have the added benefit of lowering cardiovascular risk. These therapies are an active area of research and may become an essential component of managing the dual burden of liver fibrosis and atherosclerosis in NAFLD patients [60]. Multidisciplinary care: Comprehensive management of individuals with NAFLD and the associated risk of liver fibrosis and atherosclerosis often requires a multidisciplinary approach. Collaboration between hepatologists, cardiologists, endocrinologists, and other specialists is essential for addressing the complexity of these conditions. Multidisciplinary care ensures that patients receive tailored interventions and ongoing monitoring to effectively manage their unique risk profiles [61].

Clinical management and prevention

Lifestyle Modifications and Dietary Interventions

Weight management: Achieving and maintaining a healthy weight is paramount for individuals with NAFLD. Weight loss, achieved through caloric restriction and increased physical activity, is a cornerstone of therapy. Gradual and sustainable weight loss strategies are recommended to reduce liver fat, improve insulin sensitivity, and lower the risk of atherosclerosis-related events [62].

Healthy diet: Encouraging a heart-healthy diet is essential for individuals with NAFLD, liver fibrosis, and subclinical atherosclerosis. A balanced diet should emphasize consuming fruits, vegetables, whole grains, lean proteins (such as poultry, fish, and legumes), and foods low in saturated and trans fats. This dietary approach can address multiple aspects of health, including lipid profiles, glycemic control, and liver fat accumulation [63].

Portion control: Monitoring portion sizes prevents overeating and promotes weight loss and glycemic control. Awareness of portion sizes can help individuals avoid excessive caloric intake and maintain a healthy weight [64].

Physical activity: Regular physical activity improves insulin sensitivity, reduces liver fat content, and enhances cardiovascular fitness. Combining aerobic exercises (such as brisk walking, swimming, or cycling) and resistance training (weight lifting) can be particularly beneficial. Exercise should be tailored to individual fitness levels and gradually increase over time [65].

Alcohol abstinence: For individuals with NAFLD, it is imperative to abstain from alcohol consumption, as even moderate alcohol intake can exacerbate liver damage. Eliminating alcohol is essential to prevent further harm to the liver and support liver regeneration [66].

Smoking cessation: Smoking is a well-established risk factor for cardiovascular disease. Smoking cessation is a critical component of lifestyle modification to reduce the risk of atherosclerosis-related complications. Support and resources for smoking cessation should be offered to individuals who smoke [67].

Pharmacological Approaches for NAFLD, Fibrosis, and Atherosclerosis

Pharmacotherapy for NAFLD: Although no FDA-approved medications are designated explicitly for NAFLD, ongoing research explores several potential options. Notable candidates include vitamin E, pioglitazone, and obeticholic acid. Vitamin E, a potent antioxidant, has effectively reduced liver inflammation in some individuals with NAFLD. However, its use necessitates careful consideration due to the potential risks associated with high doses. Pioglitazone, an insulin-sensitizing medication, has exhibited promise in improving liver enzyme levels, glycemic control, and histological features of NAFLD in select patients. Obeticholic acid, an FXR agonist, is currently undergoing clinical trials to treat NASH, a more severe form of NAFLD. Preliminary findings suggest its potential to reduce liver fat and inflammation [68].

Antifibrotic therapies: Recognizing the pivotal role of liver fibrosis in the progression of NAFLD, ongoing research endeavors are focused on developing antifibrotic agents tailored to the specific needs of NAFLD patients. These therapies aim to halt or reverse fibrosis, representing a critical aspect of NAFLD management. Several promising drugs are in various stages of clinical development, offering hope for more effective treatments that can address liver fibrosis directly [69].

Cardiovascular medications: Individuals confronting the dual burden of NAFLD and atherosclerosis may benefit from cardiovascular medications designed to mitigate their heightened cardiovascular risk. This pharmacological approach may encompass several categories of medications, including statins to manage dyslipidemia commonly associated with both conditions, anti-hypertensive drugs to control high blood pressure, and antiplatelet agents like aspirin to reduce the risk of clot formation and cardiovascular events. Tailoring the choice of medications, dosages, and treatment regimens to each patient's unique clinical profile and risk factors is crucial to optimizing outcomes [70].

Multidisciplinary Care and Patient Education

Multidisciplinary Care: The complexity of NAFLD and its associated complications calls for a coordinated effort among healthcare professionals, including hepatologists, cardiologists, endocrinologists, dietitians, and other specialists. Each multidisciplinary team member brings unique expertise, contributing to a comprehensive understanding of the patient's overall health and addressing specific aspects of their condition. Regular interdisciplinary meetings and consultations facilitate the exchange of information, allowing healthcare providers to tailor treatment plans to each patient's unique needs. For example, hepatologists can focus on liver-specific issues such as fibrosis assessment and management, while cardiologists can address cardiovascular risk factors and prevention strategies. This collaborative approach optimizes patient care and carefully manages potential treatment interactions [61].

Patient education: Empowering patients with knowledge about NAFLD, liver fibrosis, and atherosclerosis is a cornerstone of effective management. Patients should be well-informed about the nature of their conditions, the factors contributing to their health challenges, and the importance of adhering to prescribed treatments and lifestyle recommendations. Patient education is crucial in promoting active engagement in one’s healthcare. Patients should understand the significance of maintaining a healthy weight, following dietary guidelines, engaging in regular physical activity, and, if applicable, adhering to medication regimens. Moreover, patients should know the importance of attending regular followup appointments to monitor disease progression and treatment efficacy. With this knowledge, individuals can take proactive steps to manage their conditions effectively, reduce risks, and improve their long-term health outcomes [71].

Prevention Strategies and Public Health Implications

Early detection: Early identification of NAFLD and subclinical atherosclerosis is paramount for timely intervention. Routine screening, especially in high-risk populations such as those with obesity, diabetes, or metabolic syndrome, can enable healthcare providers to identify these conditions at an earlier, more treatable stage. Early detection provides an opportunity to implement lifestyle modifications and pharmacological interventions that can slow or even reverse disease progression [72].

Public health campaigns: Public health initiatives should prioritize raising awareness of the risks associated with obesity, unhealthy dietary habits, and sedentary lifestyles, which are significant contributors to NAFLD, liver fibrosis, and atherosclerosis. Comprehensive public health campaigns can educate individuals about maintaining a healthy weight, engaging in regular physical activity, and adopting heart-healthy dietary practices. These campaigns can also emphasize the value of regular health check-ups to monitor and manage risk factors effectively [73].

Policy changes: Policy-level interventions are essential to create environments that promote healthier lifestyles and improve access to healthcare services. Policy changes may include implementing regulations that reduce the availability and marketing of unhealthy foods, increasing opportunities for physical activity in communities, and expanding access to healthcare, particularly for underserved populations. These policy shifts can contribute to the prevention and management of NAFLD, liver fibrosis, and atherosclerosis on a broader scale [74].

Research and innovation: Ongoing research into the pathophysiology of NAFLD, liver fibrosis, and atherosclerosis is critical for advancing our understanding of these conditions and developing more effective prevention and treatment strategies. Scientific innovation can lead to the discovery of novel therapeutic targets, biomarkers for early detection, and interventions that address the root causes of these diseases. Public and private investments in research are essential to reduce the burden of NAFLD, liver fibrosis, and atherosclerosis on public health [75].

Future directions and research gaps

Emerging Research Areas in NAFLD, Fibrosis, and Atherosclerosis

Genetics and precision medicine: Research into the genetic underpinnings of NAFLD, fibrosis, and atherosclerosis is gaining momentum. Identifying specific genetic variants associated with susceptibility to these conditions can enable the development of precision medicine approaches. Tailoring interventions based on an individual's genetic profile may lead to more effective and personalized treatments and targeted prevention strategies for those at higher genetic risk.

Microbiome research: The gut microbiome's role in metabolic diseases, including NAFLD, is a burgeoning study area. Investigating how alterations in the gut microbiome contribute to liver fat accumulation and inflammation can provide insights into novel therapeutic approaches. Moreover, understanding how the gut microbiome impacts systemic inflammation and its potential influence on atherosclerosis development adds another layer of complexity to these interconnected conditions.

Epigenetics: Epigenetic modifications, such as DNA methylation and histone acetylation, are crucial in regulating gene expression. Research into epigenetic changes associated with NAFLD, fibrosis, and atherosclerosis can uncover mechanisms underlying disease development and progression. Targeting these epigenetic modifications may offer new avenues for intervention and treatment.

NASH subtypes: NASH is a severe form of NAFLD with varying clinical presentations. Emerging research is focusing on identifying distinct NASH subtypes with different pathophysiological mechanisms. Recognizing these subtypes may pave the way for tailored therapies that address the specific drivers of NASH in individual patients. This approach could improve treatment efficacy and patient outcomes.

Innovative Diagnostic and Therapeutic Approaches

Non-invasive biomarkers: The development of more accurate and specific blood-based biomarkers is a promising avenue of research. These biomarkers can provide non-invasive assessments of liver fibrosis and atherosclerosis, reducing the need for invasive procedures like liver biopsy and improving risk stratification. Advanced biomarkers may also offer insights into disease progression and response to treatment, enabling more tailored therapeutic approaches.

Imaging advancements: Ongoing advancements in imaging technologies, including artificial intelligence (AI), are transforming how we assess liver fibrosis and atherosclerosis. AI-enhanced analysis of MRI and ultrasound can provide more detailed and quantitative information about disease severity and progression. These imaging techniques offer the potential for earlier detection and more accurate monitoring, guiding treatment decisions.

Pharmacological innovations: Research into novel medications specifically targeting NAFLD, liver fibrosis, and atherosclerosis is dynamic. These medications aim to address the underlying pathophysiological mechanisms of these conditions. Innovations in drug development may lead to more effective and targeted treatment options, improving patient outcomes. Some promising avenues include antifibrotic agents for liver fibrosis and drugs simultaneously targeting liver and cardiovascular health in individuals with NAFLD and atherosclerosis.

Combination therapies: Investigating the potential synergistic effects of combining therapies for NAFLD, liver fibrosis, and atherosclerosis is an emerging approach. Combining medications or interventions that target multiple aspects of these interconnected conditions may lead to more comprehensive and effective treatment strategies. This approach recognizes the complex nature of these diseases and aims to address multiple contributing factors simultaneously.

Long-Term Outcomes and Follow-up Studies

Natural history studies: Longitudinal studies that track NAFLD patients over an extended period are essential for unraveling the natural history of the disease. These studies can provide insights into how NAFLD progresses, including the rate of fibrosis development and its impact on liver and cardiovascular health. Long-term followup can also help identify factors that influence disease trajectory and outcomes.

Survivorship research: As treatments for NAFLD, liver fibrosis, and atherosclerosis improve, it becomes increasingly important to study survivorship. Survivorship research focuses on understanding the long-term health and quality of life outcomes in individuals who have successfully managed these conditions. This research can shed light on the effectiveness of various interventions, including lifestyle modifications and pharmacological treatments, in improving long-term health outcomes, reducing the risk of disease recurrence, and enhancing overall well-being.

Policy and Guideline Development

Standardized guidelines: Establishing standardized clinical guidelines for managing NAFLD is essential. These guidelines should encompass recommendations for assessing liver fibrosis and cardiovascular risk. Standardization ensures healthcare providers have clear, evidence-based protocols to diagnose and manage these conditions. Consistent guidelines also promote equitable access to high-quality care.

Healthcare policy: Policymakers have a significant role in improving the healthcare landscape for individuals with NAFLD, liver fibrosis, and atherosclerosis. Implementing policies that enhance access to care, particularly for underserved populations, is vital. Policymakers can also support preventive measures, such as public health campaigns that raise awareness about these conditions and promote healthy lifestyles. These efforts can help reduce the prevalence and burden of NAFLD, liver fibrosis, and atherosclerosis on a population level.

Public health strategies: Public health strategies are essential for preventing and managing these interconnected conditions. Public health campaigns can educate the public about the risks associated with obesity, unhealthy diets, and sedentary lifestyles. These campaigns can also emphasize the importance of regular health check-ups and early intervention. Public health initiatives are critical in reducing disease burden and improving population health.

Healthcare integration: Another important policy consideration is encouraging healthcare systems to integrate liver and cardiovascular health assessments. Integrated care models can facilitate early detection and intervention for individuals at risk of NAFLD, fibrosis, and atherosclerosis. This approach recognizes the interplay between these conditions and ensures that patients receive comprehensive and coordinated care.

## Conclusions

In conclusion, this comprehensive review has illuminated the intricate relationship between NAFLD, liver fibrosis, and subclinical atherosclerosis. It has underscored the critical importance of recognizing these conditions as interconnected facets of a systemic disease process. Once considered benign, NAFLD is a harbinger of more severe liver-related complications and heightened cardiovascular risk. Managing liver fibrosis and atherosclerosis is paramount for improving patient outcomes, reducing cardiovascular risk, and fostering a holistic approach to healthcare. As we move forward, healthcare professionals must prioritize early detection and multidisciplinary collaboration, researchers should continue advancing our understanding, and patients should be empowered to advocate for their health. By addressing this complex interplay, we have the potential to enhance the well-being of individuals affected by NAFLD and pave the way for a healthier future.
